# Deodorization of Tuna Peptides by Hydrogen Peroxide Oxidation

**DOI:** 10.3390/molecules31040726

**Published:** 2026-02-20

**Authors:** Huaye Tong, Jiongfeng Li, Minjie Zheng, Xingya Fan, Wenbing Yuan, Jiangshang Su, Daofei Lv, Feng Xu, Xin Chen

**Affiliations:** School of Environment and Chemical Engineering, Foshan University, Foshan 528000, China; 19117721752@163.com (H.T.); ljf999888202205@163.com (J.L.); m18138527256@163.com (M.Z.); 17686248849@163.com (X.F.); hnyuanwb@126.com (W.Y.); s18239171275@126.com (J.S.); fengxu@fosu.edu.cn (F.X.)

**Keywords:** tuna peptide, fishy odor, hydrogen peroxide oxidation, cosmetics

## Abstract

Tuna peptides possess significant bioactivity but are limited by their persistent fishy odor. This study employed mild oxidation with medical-grade hydrogen peroxide (3% H_2_O_2_) to deodorize tuna peptides. The optimal parameters determined through single-factor and orthogonal experiments were 798 mmol/L H_2_O_2_, 35 °C, and 20 min. Under these conditions, the sensory score decreased markedly from 5 (very strong odor) to 2.48 (slight odor). Solid-phase microextraction and gas chromatography/mass spectrometry (SPME-GC/MS) analysis confirmed the complete removal of key odorants such as octanal and heptanal, along with a 44.8–54.7% reduction in other volatile compounds. Importantly, the treated peptides retained substantial antioxidant activity, with 2,2-azinobis (3-ethylbenzothiazoline-6-sulfonic acid) (ABTS) and 2,2-diphenyl-1-picrylhydrazyl (DPPH) radical scavenging rates of 91.5% and 78.3%, respectively. Successful incorporation of the deodorized peptides into a moisturizer demonstrated effective and lasting odor reduction. The proposed method offers an efficient, mild, and industrially viable strategy to expand the application of tuna peptides in functional cosmetics and foods.

## 1. Introduction

Tuna (Osteichthyes: Perciformes: Scombridae) is a general term for several genera of fish with a corselet (significantly enlarged scales on the thoracic area and anterior to the lateral line). Tuna are widely distributed in the vast waters of the tropical, subtropical and temperate regions of the Atlantic, Pacific and Indian Oceans [1,2]. It is an important commercial edible fish, with strong reproductive ability, high economic value, low fat, and high protein. It is one of the three most nutritious fish recommended by the World Health Nutrition Organization [3,4,5]. Tuna peptides are typically prepared by pyrolyzing, chemically degrading, or enzymatically hydrolyzing macromolecular proteins in tuna [6,7]. Tuna peptides exhibit a wide range of biological functions, including antioxidant, immune regulation, uric acid lowering, anti-fatigue, anti-osteoporosis, antibacterial, and anti-inflammatory activities, and weight loss [8,9,10,11,12]. Among these, their antioxidant properties are of particular note, rendering them promising candidates as functional ingredients in cosmetic and skincare formulations, where they could help protect the skin from oxidative stress [13]. However, the particular fishy odor of tuna peptides limits the application and development of tuna peptides in the fields of cosmetics [14].

The main fishy odor substances in tuna peptide products include hexanal, heptanal, nonanal, 2-hexenal, 1-octen-3-ol, and trimethylamine [15]. These compounds primarily originate from the oxidation of unsaturated fatty acids and the degradation of trimethylamine oxide during processing and storage. Their low odor thresholds and lipophilic nature enable them to form stable complexes by binding strongly to the hydrophobic regions of peptides and proteins, which renders their removal by simple physical methods particularly challenging [16]. The commonly used deodorization methods for small-molecule peptides are roughly divided into four categories: physical, biological, chemical, and combined deodorization [17,18,19,20]. Liu et al. [15] tried to eliminate the fishy odor substances in the enzymatic hydrolyzate of tuna by physical deodorization and found that the optimum conditions for deodorization were 3% activated carbon, pH = 3.0, temperature = 70 °C, and time = 60 min, which reduced the volatile substances by 85.39%. Pan et al. [21] tried to remove the odor of tiger puffer skin gelatin by biological deodorization, and found that aldehyde and ester volatile substances were reduced by 82.7% and 94.0%, respectively, with 1% yeast, but alcohol volatile substances increased by 10.2%. Huang et al. [20] used a combined deodorization method to remove the fishy odor from cod skin and found that the optimum deodorization process was fermentation with 1.5% yeast powder for 90 min at pH 5.0 and 35 °C, followed by a reaction with 2.0% powdered activated carbon for 70 min at 40 °C. Yeast powder is used for three reasons: (1) its loose structure can adsorb fishy odor substances; (2) enzymes in the yeast react with the fishy odor substances (e.g., ketones and aldehydes) to convert them into odorless substances; (3) some intermediate products of fermentation may mask the fishy odor.

Compared with other deodorization methods, chemical deodorization has the advantages of a large processing capacity, high deodorization efficiency, and simple operation, and therefore is considered to be promising for industrial application [22]. Qiu et al. [23] extracted fishy odor substances from the enzymatic hydrolysate of mackerel, using ether for deodorization. After three extractions with ether, the sensory score of the hydrolysate decreased from 9.0 to 4.5, indicating a mediocre deodorization effect. Moreover, it can easily lead to organic solvent residues. Du et al. [24] found that ozone completely or partially removed fishy odor components, such as aldehydes, ketones, and alcohols, in silver carp surimi. Ozone works by producing extremely active and strongly oxidizing monatomic oxygen and hydroxyl radicals to oxidize fishy odor substances in water. The disadvantage of this method is that the oxidizing property of ozone is too strong, which easily causes protein denaturation. Among chemical oxidants, hydrogen peroxide (H_2_O_2_) presents itself as a promising alternative. It is an environmentally benign oxidant that decomposes into water and oxygen, leaving no harmful residues. Its efficacy in eliminating odors has been demonstrated in seafood. Wang et al. [25] found that using 80 mM H_2_O_2_ during the rinsing stage of skipjack tuna surimi gel preparation significantly reduced the fishy odor of the product and improved its texture and whiteness. Their molecular dynamics simulations further revealed that H_2_O_2_ treatment weakened the binding capacity of myosin to key fishy odor molecules such as octanal and 1-octen-3-ol, thereby reducing the stability of the complex. Meanwhile, Liu et al. [15] reached similar conclusions in their study on deodorizing sea cucumber peptides. By using 816.2 mmol/L medical-grade H_2_O_2_ under mild conditions (35 °C, 30 min), they successfully achieved effective deodorization. Moreover, the total nitrogen and amino acid nitrogen contents of the treated products remained stable, indicating minimal impact on the main active components. However, its application in deodorizing tuna peptides, particularly under mild conditions using low concentrations, remains underexplored. Antioxidant capacity of tuna peptides is considered one of the most important parameters related to the quality of final products. Products containing tuna peptides with higher antioxidant capacity showed better sensory quality and stability [26]. However, hydrogen peroxide with a high concentration (30%) shows a very strong oxidizing ability. Comparatively, the concentration of medical-grade hydrogen peroxide is about 3%, which is a mild antiseptic used on the skin to prevent infection of minor cuts, scrapes, and burns [27].

MnO_2_ was employed to catalytically decompose residual H_2_O_2_ after deodorization. However, since this step may introduce trace amounts of manganese into the peptide matrix, quantification of residual manganese was necessary to assess its potential impact on product safety and quality, particularly for cosmetic applications where heavy metal content is strictly regulated [28].

In this study, oxidation with medical hydrogen peroxide (mass concentration 3%) was used to deodorize tuna peptides. Sensory evaluation, GC/MS, total nitrogen determination, amino acid nitrogen determination and ABTS radical scavenging assay showed that hydrogen peroxide has a very significant deodorizing effect on tuna peptides. We also examined the effects of hydrogen peroxide concentration and reaction temperature and time on the deodorization of tuna peptides to identify the optimal conditions of deodorization by hydrogen peroxide oxidation.

## 2. Results

### 2.1. Single-Factor Experiments

To observe the persistence of the deodorizing effect over storage time, tuna peptide samples were sensory evaluated by 10 evaluators immediately, and 2 and 4 days after deodorization.

#### 2.1.1. Impact of Hydrogen Peroxide Concentration on Deodorization

**Figure 1:**
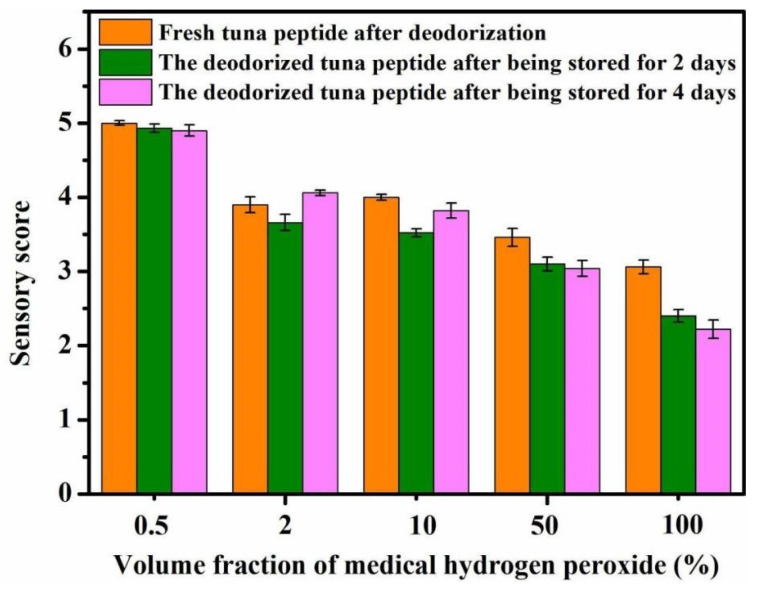
Effect of H_2_O_2_ concentration on sensory scores of tuna peptides during storage (0, 2, 4 days).

As shown in Figure 1, the concentration of medical hydrogen peroxide directly affects the sensory score of tuna peptides. The lower the concentration of hydrogen peroxide, the higher the sensory score, and the higher the concentration, the greater the deodorizing effect. A concentration of 100% hydrogen peroxide achieved a score of 1.75, which indicates a very slight fishy odor.

#### 2.1.2. Impact of Reaction Time on Deodorization

**Figure 2:**
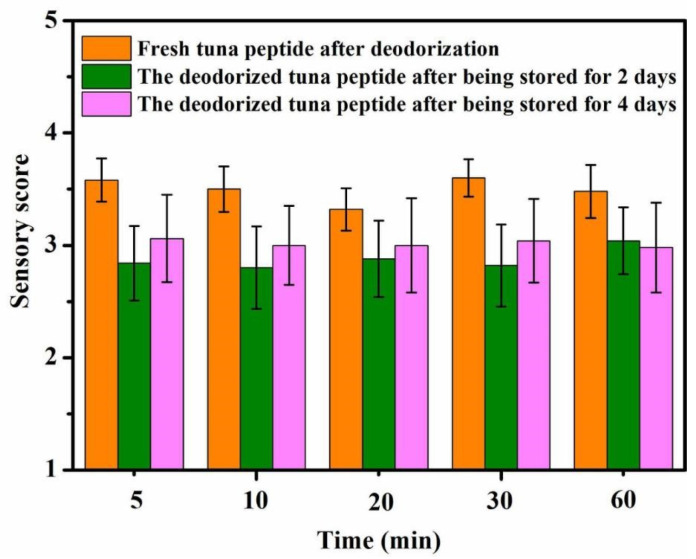
Effect of reaction time on sensory scores of tuna peptides during storage (0, 2, 4 days).

As shown in Figure 2, a long period of immersion in hydrogen peroxide does not improve deodorization. The fishy odor substances reacted completely with hydrogen peroxide 5 min after its addition. At this time, the sensory score was approximately 2.7, indicating a slight fishy odor. It can thus be seen that hydrogen peroxide oxidation has high reaction efficiency for deodorization.

#### 2.1.3. Impact of Reaction Temperature on Deodorization

**Figure 3:**
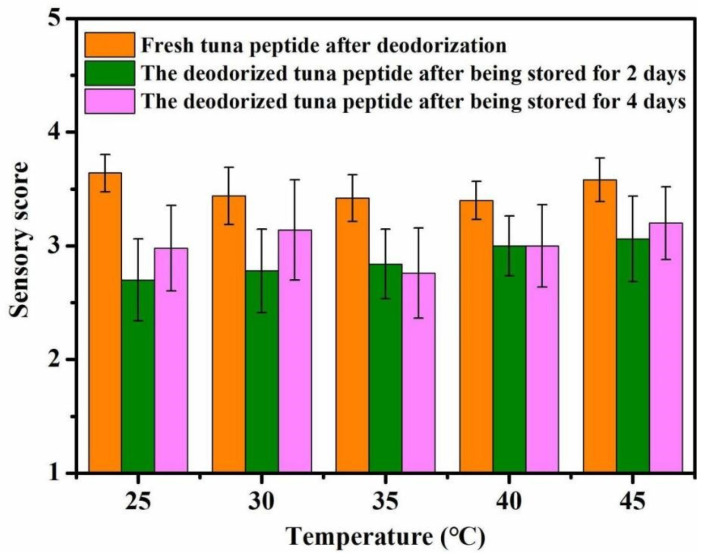
Effect of reaction temperature on sensory scores of tuna peptides during storage (0, 2, 4 days).

As shown in Figure 3, reaction temperature has little effect on the deodorization of tuna peptides. Overall, the optimal deodorizing effect was achieved at 25 °C. The deodorizing effect decreased with increasing temperature.

### 2.2. Orthogonal Analysis of Factors Affecting the Deodorization of Tuna Peptides

To ensure the accuracy of the results, the experiment was repeated three times for each factor level, and the mean of the three measurements was used for orthogonal analysis.

According to orthogonal experimental principles, the range value (R) reflects the relative influence of each factor on the response variable, with larger R values indicating greater impact. The R-value for each factor was calculated by summing the results at each level (K), averaging to obtain mean values (k), and then determining the difference between the maximum and minimum k values. This approach was employed to identify the relative importance of H_2_O_2_ concentration, reaction time, and reaction temperature on the deodorization efficiency of tuna peptides.

#### 2.2.1. Range Analysis

According to the F distribution critical value table, F_0.05_ (4,20) = 2.87 < F_A_, P_A_ < 0.05 (Table 1), indicating that factor A has a significant influence and other factors have no significant influence [29]. As can be seen from Table 2, the factors influencing deodorization are, in descending order of importance: hydrogen peroxide concentration, reaction temperature, and reaction time. Based on the above analysis, the hydrogen peroxide concentration is a significant influencing factor, and the optimal level combination is A_5_B_3_C_3_, which means 100% hydrogen peroxide for 20 min at 35 °C. However, A_5_B_3_C_3_ is not among the 25 options of the orthogonal experiment. Therefore, a confirmatory experiment was required.

#### 2.2.2. Confirmatory Experiment

As shown in Table 1, A_5_B_3_C_2_ had the highest total score of 2.53, whereas the optimal level combination was A_5_B_3_C_3_. Accordingly, a confirmatory experiment was performed. As can be seen from the results given in Table 3, the sensory score of the optimal level combination A_5_B_3_C_3_ is 2.48, which is similar to that of A_5_B_3_C_2_.

### 2.3. GC-MS Results

The untreated tuna peptides and those treated with 798 mmol/L hydrogen peroxide at 35 °C for 20 min were analyzed by GC-MS (Figures S1 and S2). Their volatile profiles are summarized in Table 4. Notably, the treatment led to the complete disappearance of caprylaldehyde and heptanal—two potent fishy odorants. Concurrently, the peak areas of other aldehydes (capraldehyde, nonanal) and ketones (e.g., 6,10-dimethyl-5,9-undecadien-2-one) were reduced. This significant shift in the volatile profile, particularly the removal of key aldehydes, correlates with the observed reduction in sensory fishy odor scores.

### 2.4. Total Nitrogen and Amino Acid Nitrogen in Tuna Peptides

The total nitrogen and amino acid nitrogen content of the untreated tuna peptides and those treated with 798 mmol/L hydrogen peroxide at 35 °C for 20 min were determined. The results are shown in Table 5. After treatment, the total nitrogen content was decreased by 9.7%, and the amino acid nitrogen content was increased by 41.9%. The decrease in total nitrogen coupled with the increase in amino acid nitrogen suggests that hydrogen peroxide oxidation likely induced cleavage of some peptide bonds.

### 2.5. Cosmetic Test Results

To observe the acceptability of the fishy odor of deodorized tuna peptides in a moisturizer (Table S1), moisturizer samples containing 1% untreated tuna peptides and 1% tuna peptides treated with 798 mmol/L hydrogen peroxide at 35 °C for 20 min were sensory evaluated three times: immediately, and 2 and 4 days after the addition of tuna peptides.

**Figure 4:**
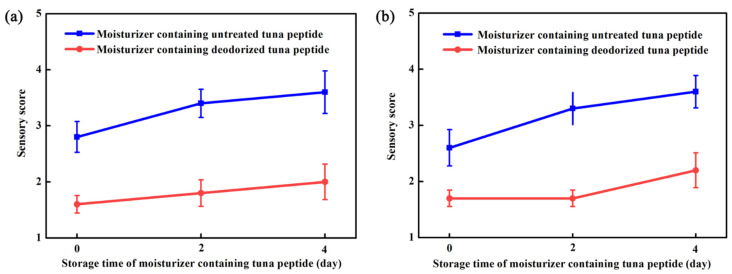
Moisturizer formulations containing 1% (*w*/*w*) untreated or deodorized tuna peptides were evaluated immediately (day 0) and after 2 and 4 days of storage. (**a**) Not applied on the skin and (**b**) evenly applied on the skin.

Figure 4a,b show that treatment with hydrogen peroxide had a significant deodorizing effect on the moisturizer, both in paste form and after application. The moisturizer containing untreated tuna peptides in paste form was yellow, whereas that containing treated tuna peptides was light yellow and had almost no fishy odor. The spreadability, wetness, and stickiness of the moisturizer samples containing untreated and treated tuna peptides in paste form and the softness and glossiness after application were the same as the blank control.

### 2.6. Antioxidant Activity Assessment Results

To evaluate the impact of hydrogen peroxide treatment on the antioxidant properties of tuna peptides, their free radical scavenging capacities were determined using the ABTS and DPPH assays. As summarized in Tables S2 and S3, the untreated tuna peptides exhibited excellent antioxidant activity, with an ABTS radical scavenging rate of 99.2 ± 1.5% and a DPPH radical scavenging rate of 85.7 ± 2.1%. Following deodorization under the optimized conditions (798 mmol/L H_2_O_2_, 35 °C, 20 min), the peptides retained an ABTS scavenging rate of 91.5 ± 1.8% and a DPPH scavenging rate of 78.3 ± 2.4%. Although a slight reduction in scavenging activity was observed for both radicals after treatment, the core bioactive function was largely preserved.

### 2.7. Quantitative Determination of Residual Hydrogen Peroxide

Residual hydrogen peroxide was undetectable by both analytical approaches. The semi-quantitative test-strip assay (detection limit = 25 mg L^−1^) yielded negative results for all treated samples (Figure S5). Subsequent quantification via the titanium-oxysulfate spectrophotometric method (LOD = 0.3 μg mL^−1^, LOQ = 1.0 μg mL^−1^) confirmed that the residual H_2_O_2_ concentration in every post-treatment sample was below the detection limit of the validated assay (Figure S8). These data collectively demonstrate the complete and effective removal of hydrogen peroxide after the MnO_2_-quenching step.

### 2.8. ICP-OES Results

The analytical data confirm that the introduction of manganese into the peptide matrix is attributable to the step employing manganese dioxide for the catalytic decomposition of residual hydrogen peroxide. The manganese content exhibited a 330-fold increase following treatment, rising from 0.04 mg/L to 13.21 ± 1.72 mg/L. Although the treated peptide solution demonstrated a marked elevation in manganese concentration, its practical exposure risk can be substantially mitigated through formulation dilution when utilized as a functional ingredient in the final product. In the present study, incorporation of the treated peptide at 1% (*w*/*w*) into the cosmetic base resulted in residual manganese levels that remain within acceptable safety thresholds.

### 2.9. LC-MS Results

A significant reduction in the relative abundance of antioxidant-characteristic residues—notably histidine, tyrosine, tryptophan, methionine, and cysteine—was observed across the peptide mixture after hydrogen peroxide treatment (Figures S6 and S7). Notably, several peptides containing characteristic antioxidant residues, including the representative peptide DFDHSCQRETPSVVAMIYPFVG, were identified to remain stable or even exhibit increased abundance post-treatment (Table S5). This observation suggests that these specific peptides may possess distinctive antioxidant stability, potentially reflecting inherent structural resistance to oxidative degradation or induction under oxidative stress conditions.

## 3. Discussion

Hydrogen peroxide exhibited significant deodorization efficacy on tuna peptides, with concentration identified as the key influencing factor. Under optimized conditions (798 mmol/L H_2_O_2_, 35 °C, 20 min), the sensory score decreased from 5.0 to 2.48. The odor reduction achieved was comparable to that reported for yeast–activated carbon combined methods, yet with a substantially shorter processing time [14].

GC–MS analysis confirmed the complete removal of key fishy aldehydes (octanal and heptanal) and a 44.8–54.7% reduction in other volatiles. Notably, the oxidation generated low-odor ketones (3-heptanone, 2-octanone), providing direct evidence for the chemical conversion of pungent aldehydes. Compared to physical adsorption, which removes volatiles via physisorption [14], H_2_O_2_ oxidation enables both removal and chemical transformation of odorants. In contrast to yeast fermentation, which may generate new odorants [21,30,31], H_2_O_2_-mediated hydroxyl radical oxidation provides more comprehensive elimination of odorous compounds.

The deodorization mechanism involves the generation of hydroxyl radicals (·OH) from H_2_O_2_ decomposition, which rapidly oxidize aldehydes, ketones, and sulfur-containing compounds into low-odor or odorless products. This radical-mediated pathway is similar to that proposed for ozone treatment of silver carp surimi [24], but with the distinct advantage of milder oxidizing conditions that minimize collateral damage to peptide structures. Indeed, the deodorization process was accompanied by only mild peptide-bond cleavage, as evidenced by a 9.7% decrease in total nitrogen and a 41.9% increase in amino nitrogen—a level of modification substantially lower than that associated with ozone treatment, which often induces extensive protein denaturation and functionality loss [24,32].

Despite this limited structural modification, the treated peptides retained strong antioxidant activity, with ABTS and DPPH radical scavenging rates of 91.5% and 78.3%, This level of bioactivity preservation is notably higher than that reported for harsher chemical methods. For instance, ozone treatment of aquatic products has been associated with significant protein denaturation and loss of functional properties [24,32], while solvent extraction methods carry inherent risks of toxic residue retention and potential degradation of peptide integrity [23,33]. LC–MS analysis further revealed that several antioxidant-rich peptides, including the representative sequence DFDHSCQRETPSVVAMIYPFVG, remained stable or even increased in abundance post-treatment, indicating selective protection of functional peptide sequences under mild oxidative conditions. This selective stability suggests that these peptides may possess inherent structural resistance to oxidative degradation, contributing to the overall preservation of bioactivity.

Residual H_2_O_2_ was effectively eliminated via MnO_2_ quenching, with post-treatment levels below the detection limit. Although manganese was introduced during this step, its concentration in the final cosmetic formulation (at 1% peptide incorporation) remained within acceptable safety thresholds. The method is operationally simple, leaves no harmful residues, and is readily scalable. Successful incorporation of the deodorized peptides into a moisturizer confirmed its practical applicability and sensory acceptability.

In summary, mild H_2_O_2_ oxidation offers an efficient deodorization strategy for tuna peptides, combining effective odor removal with preservation of antioxidant activity. The mechanism involves hydroxyl radical-mediated oxidation of odorants coupled with selective protection of functional peptides, offering advantages over physical adsorption (mere transfer of odorants), biological fermentation (potential generation of new off-odorants), and harsh chemical treatments (extensive protein damage).

## 4. Materials and Methods

### 4.1. Materials

Tuna peptides (with a peptide mass fraction of 85%) were purchased from Liaoning Tai’ai Peptide Biotechnology Co., Ltd. (Shenyang, China). The purchased tuna peptides were prepared from yellowfin tuna as the raw material. Tuna peptides exhibited a weight distribution: >2 kDa (4.33%), 1–2 kDa (13.32%), 0.5–1 kDa (30.63%), and <0.5 kDa (51.72%). Hydrogen peroxide (mass fraction of about 3%) was obtained from Guangdong Hengjian Pharmaceutical Co., Ltd. (Foshan, China). The hydrogen peroxide test strips were purchased from MACHEREY-NAGEL (Düren, Germany). All other reagents used were of analytical grade.

Instruments used included a gas chromatograph–mass spectrometer (GC-MS; 7890A-5975C) (Agilent Technologies, Santa Clara, CA, USA), a liquid chromatography mass spectrometer (LC-MS; Easy nLC-Q-Exactive HF-X) (Thermo Fisher Scientific, Waltham, MA, USA), a inductively coupled plasma optical emission spectrometer (ICP-OES; 720ES) (Agilent Technologies, Santa Clara, CA, USA), a water bath constant temperature magnetic stirrer (DZKW-S-8) (Beijing Yongguangming Medical Instrument Co., Ltd., Beijing, China), a Kjeldahl nitrogen analyzer (K9860) (Jinan Hanon Instruments Co., Ltd., Jinan, China), a freeze-dryer (Labconco, Kansas City, MO, USA), a Midea refrigerator (BCD-112CM) (Midea Group, Foshan, China), a MAPADA ultraviolet-visible spectrophotometer (V-1100D) (Shanghai MAPADA Instruments Co., Ltd., Shanghai, China), a high-speed disperser (IKA Works, Staufen, Germany), and a Sartorius electronic balance (BSA224S-CW) (Sartorius AG, Göttingen, Germany).

### 4.2. Determination and Dilution of Hydrogen Peroxide

A sodium oxalate standard solution was prepared by dissolving 1.6841 g in distilled water. The solution was used to standardize potassium permanganate via triplicate titrations, which consumed 22.5, 22.5, and 22.4 mL, respectively, yielding a standardized concentration of 0.22 mol/L. Subsequently, the standardized potassium permanganate was employed to titrate medical-grade hydrogen peroxide. The titrations required 14.3, 14.3, and 14.2 mL in three repeated measurements, resulting in a determined hydrogen peroxide concentration of 798 mmol/L, corresponding to a mass fraction of 2.7% [34]. Hydrogen peroxide solutions with volume fractions of 100% (798 mmol/L), 50% (399 mmol/L), 10% (79.8 mmol/L), 2% (15.96 mmol/L), and 0.5% (3.99 mmol/L) were prepared for single-factor experiments.

### 4.3. Deodorization of Tuna Peptides

Tuna peptide powder (1 g) was dissolved in 2 mL of deionized water in a beaker. The tuna peptide solution was treated with hydrogen peroxide [35] at five designated concentrations (0.5%, 2%, 10%, 50%, or 100%) by thoroughly mixing 1 mL of the solution with 3 mL of H_2_O_2_. The reaction was conducted under controlled conditions in a constant-temperature water bath. A full-factorial experimental design was implemented to systematically evaluate all combinations of three key parameters: hydrogen peroxide concentration (five levels: 0.5%, 2%, 10%, 50%, and 100%), incubation temperature (five levels: 25, 30, 35, 40, and 45 °C), and treatment time (five levels: 5, 10, 20, 30, and 60 min). Upon completion of the oxidation reaction, a trace amount of manganese dioxide was introduced into each sample to catalytically decompose any residual hydrogen peroxide [36].

### 4.4. Sensory Evaluation

Ten panelists (five from inland provinces and five from coastal provinces; age range: 18–60 years; five men and five women) performed a descriptive sensory analysis of the samples at room temperature. All panelists were healthy adult volunteers who were fully informed about the nature of the samples (food-grade tuna peptides) and the experimental procedure, and provided informed consent prior to participation. The panelists underwent one week of training prior to the evaluation, as per the procedure outlined in Chen et al. [36]. Training protocols included familiarization with the “fishy odor” attribute using a defined set of chemical references (e.g., heptanal and nonanal solutions at specified concentrations) as well as repeated exposure to tuna peptide samples spanning the full intensity range. Samples (approximately 10 mL) were dispensed into identical opaque containers and presented in a randomized order. Panelists were instructed to cleanse their palates with water and unsalted crackers between sample evaluations. The filtrate was sensory evaluated using a 5-point scale (1, no fishy odor; 2, slight fishy odor; 3, moderate fishy odor; 4, heavy fishy odor; 5, very heavy fishy odor). The sensory evaluation criteria are shown in Table 6. Water without adding tuna peptides was used as a standard for no fishy odor and untreated tuna peptides for heavy fishy odor. All samples, including controls, were evaluated in triplicate by each panelist across three independent sessions. Statistical analysis was performed using one-way analysis of variance (ANOVA) with SPSS software (version 26.0, IBM Corp., Armonk, NY, USA), with *p* < 0.05 considered statistically significant [37].

A composite score S was obtained according to the following equation (Equation (1)):

(1) $$S\;=\;\sum \frac{B_{i}}{n}$$

where S is the composite score from the sensory evaluation; Bi is the score given by the panelists; and n is the total number of sensory evaluation panelists.

### 4.5. Single-Factor Experimental Design

#### 4.5.1. Hydrogen Peroxide Concentration

At a reaction time of 20 min at 25 °C, the deodorizing effects of hydrogen peroxide concentrations of 0.5%, 2%, 10%, 50%, and 100% on tuna peptides were investigated. For the experiments of studying the deodorizing effects of hydrogen peroxide concentrations, the concentration of hydrogen peroxide value ratio/peptide was 3.99, 15.96, 79.8, 399, and 798 mmol/(g·L), respectively.

#### 4.5.2. Reaction Time

At a hydrogen peroxide concentration of 10% at 25 °C, the deodorizing effects of reaction times of 5, 10, 20, 30, and 60 min on tuna peptides were investigated.

#### 4.5.3. Reaction Temperature

At a hydrogen peroxide concentration of 10% and a reaction time of 20 min, the deodorizing effects of reaction temperatures of 25, 30, 35, 40, and 45 °C on tuna peptides were investigated.

### 4.6. Orthogonal Experimental Design for Deodorization

To systematically optimize the deodorization process, a three-factor, five-level orthogonal experimental design was implemented. The design investigated the effects of hydrogen peroxide concentration (Factor A), reaction time (Factor B), and reaction temperature (Factor C) on the deodorization efficiency of tuna peptides. The specific level settings are presented in Table 7. The experimental data were statistically analyzed using Analysis of Variance (ANOVA) and Tukey’s multiple comparison test (*p* < 0.05) via SPSS software. This analysis aimed to determine the significance of each factor, their order of influence, and ultimately identify the optimal deodorization conditions.

### 4.7. SPME-GC/MS Determination

HP-5MS (30 m × 0.25 mm × 0.25 μm) was used as the separation column. The injection temperature was 250 °C, the carrier gas was He, and the flow rate was 1.0 L/min. The sample was splitless-injected. The initial column temperature was kept at 40 °C for 2 min, then raised to 160 °C at 4 °C/min, again raised to 250 °C at 8 °C/min, and maintained at that temperature for 10 min. The mass spectrometry (MS) conditions were: solvent cut time 1 min, electron impact (EI) ion source, ion source temperature 230 °C, ionization voltage 70 eV, scan mass range 30–500 *m*/*z*, interface temperature 280 °C, and quadrupole temperature 150 °C.

The untreated and treated samples of tuna peptides were analyzed by GC-MS. To investigate the content of volatile substances, the peak areas of the main volatile substances in the samples were determined by observing the characteristic peaks of the ions. All samples were analyzed in randomized order. Procedural blanks (using the same solvents and undergoing identical extraction and derivatization steps without any tuna peptide) were included in each analytical batch to monitor for background contamination.

### 4.8. Determination of Total Nitrogen and Amino Acid Nitrogen

#### 4.8.1. Total Nitrogen

First, 0.4 g of the untreated and treated samples of tuna peptides were each transferred to a digestion tube (designated as tubes 1 and 2), followed by the addition of 0.3 g of copper sulfate pentahydrate and 3 g of potassium sulfate as catalysts, respectively. Next, 10 mL of concentrated sulfuric acid was drawn from each of the two digestion tubes. A small funnel was placed on the top of each tube. The digestion tubes were then heated using a graphite block digester according to the following program: 170 °C for 30 min, 270 °C for 30 min, 400 °C for 90 min, and cooling to room temperature.

Prepared sodium hydroxide solution, boric acid solution, and deionized water were added to the Kjeldahl nitrogen analyzer and preheated. After preheating, Erlenmeyer flask 1 was collected and connected to an absorption tube. The contents were diluted with 10 mL of water. Then, 25 mL of boric acid solution and 65 mL of 40% sodium hydroxide solution were added. The mixture was distilled for 5 min and rinsed with 10 mL of water. After distillation, the liquid in Erlenmeyer flask 1 was titrated with 0.1 mol/L hydrochloric acid standard solution. Similarly, the treated samples were distilled and titrated following the same procedure. The volume of hydrochloric acid standard solution consumed (VHCl) was recorded for each sample and used to calculate the total nitrogen content [38].

#### 4.8.2. Amino Acid Nitrogen

First, 1.0 g of the untreated and treated tuna peptides were each dissolved in 65 mL of deionized water, and the pH was adjusted to 8.2. Then, pH = 8.2 neutral formaldehyde solution was added, the solution stirred and titrated against 0.1 mol/L sodium hydroxide solution. The endpoint of the titration was reached when the pH became 9.2 and remained unchanged for 30 s after the addition of the last drop of sodium hydroxide (NaOH) solution. At this point, the amount of NaOH solution used was read off the burette scale was recorded. The volume of NaOH standard solution consumed (V_NaOH_) was the amount needed to raise the pH of the enzymatic hydrolysate to 9.2 after adding the neutral formaldehyde solution. A blank control was prepared in the same way, and the volume of NaOH was recorded [39].

### 4.9. Fishy Odor Test of Tuna Peptide Cosmetics

The untreated and well-deodorized samples of tuna peptides were each added to a moisturizer (the tuna peptides make up 1% of the total raw materials of the moisturizer, the detailed formulation of the moisturizer base is provided in Table S1) to test the deodorizing effect of hydrogen peroxide on the actual product. Sensory evaluation of the fishy odor was done of the moisturizer, both in paste form and after spreading.

### 4.10. Determination of Antioxidant Activity

#### 4.10.1. Sample Preparation

Freeze-dried samples of the original and hydrogen peroxide-treated tuna peptides were precisely weighed (0.035 g each) and dissolved in 10 mL of deionized water to obtain stock solutions at a concentration of 3.5 mg/mL.

#### 4.10.2. ABTS Radical Scavenging Assay

The ABTS radical cation stock solution was generated by mixing equal volumes of 7.0 mmol/L ABTS aqueous solution and 2.45 mmol/L potassium persulfate solution, followed by incubation in the dark at room temperature for 12–16 h. Prior to analysis, this stock solution was diluted with deionized water to achieve an absorbance of 0.70 ± 0.02 at 734 nm. For the assay, 1 mL of the sample stock solution (or deionized water for the blank control) was combined with 3 mL of the diluted ABTS working solution. The mixture was vortexed thoroughly, allowed to react in the dark for 10 min, and the absorbance was measured immediately at 734 nm [40].

#### 4.10.3. DPPH Radical Scavenging Assay

A 0.1 mmol/L working solution of DPPH was prepared fresh in absolute ethanol. For the assay, 2 mL of the sample stock solution (or deionized water for the blank) was mixed with 2 mL of the DPPH solution. The reaction mixture was vortexed and incubated in the dark at room temperature for 30 min. Subsequently, the absorbance was measured at 517 nm [40].

The radical scavenging activity for both assays was calculated as a percentage using the following formula:

(2) $$\mathrm{Scavenging rate}\;(\%)=(\frac{A_{0}-A}{A_{0}})\times 100$$

where A_0_ represents the absorbance of the blank control, and A represents the absorbance of the sample reaction mixture. All measurements were performed in triplicate.

### 4.11. Quantification of Residual Hydrogen Peroxide

Residual hydrogen peroxide was assessed using a two-tiered analytical approach. Initial screening was conducted with commercial Quantofix Peroxide 25 test strips. a test strip was immersed in the sample solution for 1 s, withdrawn, and the developed color was compared against the provided color scale after 15 s. This method served for rapid on-site semi-quantitative evaluation [41].

For precise quantification, a spectrophotometric method based on the titanium oxysulfate reaction was employed. A 10% (*v*/*v*) titanium oxysulfate working solution was prepared. A calibration curve was constructed using hydrogen peroxide standards (0–8 μg/mL) prepared in a 0.5% untreated peptide matrix (Figure S8). For analysis, 2.00 mL of sample or standard was mixed with 1.00 mL of the colorimetric reagent. After incubation at room temperature for 15 min, the absorbance was measured at 410 nm, using a sample blank (peptide solution mixed with water) as the reference [42].

### 4.12. Quantification of Residual Manganese

The manganese content in the deodorized tuna peptide solution was determined using inductively coupled plasma optical emission spectrometry (ICP-OES). The instrument was operated under the following optimized conditions: radiofrequency power, 1.20 kW; plasma gas flow, 15.0 L/min; auxiliary gas flow, 1.50 L/min; nebulizer gas flow, 0.75 L/min; and sample uptake delay, 15 s. Quantification was performed at the characteristic manganese emission line of 257.610 nm. External calibration was conducted using a series of manganese standard solutions (0, 0.05, 0.1, 0.5, and 1.0 mg/L) prepared in 2% (*v*/*v*) nitric acid [43].

### 4.13. LC/MS Determination

#### 4.13.1. Sample Preparation

Peptide extraction was performed by homogenizing 1 mg of dried peptide or 100 µL of peptide solution with 100 µL of 0.1% trifluoroacetic acid (TFA), followed by centrifugation at 14,000× *g* for 10 min. The supernatant was subjected to ultrafiltration using a 10 kDa cutoff device (PALL) at 13,500× *g* for 10 min. Peptide concentration in the filtrate was determined spectrophotometrically, after which samples were desalted with a C18 solid-phase extraction cartridge using acetonitrile conditioning, TFA equilibration, and elution with 70% acetonitrile [44].

#### 4.13.2. Chromatographic Separation

Desalted peptides were separated on a nano-flow HPLC system (Easy nLC, Thermo Scientific) equipped with a trapping column (100 µm × 2 cm, 5 µm C18) and an analytical column (75 µm × 10 cm, 3 µm C18). Separation was achieved at 250 nL/min using a gradient of 0.1% formic acid in water (solvent A) and 0.1% formic acid in 84% acetonitrile (solvent B): 0–35% B over 50 min, 35–100% B from 50 to 58 min, and 100% B from 58 to 60 min.

#### 4.13.3. Mass Spectrometric Identification

MS analysis was conducted on a Q-Exactive HF-X mass spectrometer (Thermo Fisher Scientific) in positive ion mode over a 60 min gradient. Full MS scans (300–1800 *m*/*z*) were acquired at a resolution of 70,000 (at *m*/*z* 200) with an AGC target of 3 × 10^6^ and maximum IT of 10 ms. For MS/MS, the top 10 most intense precursors were fragmented via HCD (isolation window 2 *m*/*z*, NCE 30 eV), with MS/MS spectra collected at a resolution of 17,500 (at *m*/*z* 200), an underfill ratio of 0.1%, and an AGC target of 1 × 10^5^. Dynamic exclusion was set to 40 s.

## 5. Conclusions

In summary, this study demonstrates an efficient and mild deodorization method for tuna peptides using 3% H_2_O_2_ under optimized conditions (798 mmol/L, 35 °C, 20 min). The treatment significantly reduced fishy odor, evidenced by a lowered sensory score and the removal of key volatile aldehydes, while largely preserving antioxidant activity (91.5% ABTS and 78.3% DPPH scavenging rates). The method is advantageous due to its simplicity, use of a food-grade oxidant, mild operating conditions, and scalability. However, limitations include peptide bond cleavage and the introduction of manganese ions from the quenching step, which requires careful control in final formulations. This approach offers a practical strategy to enhance the applicability of tuna peptides in functional cosmetics and foods.

## Figures and Tables

**Table 1:** Analysis of variance results.

Source of Variance	Quadratic Sum	Degree of Freedom	Mean Square	F Values	*p* Values	Significance
A	10.588	4	2.647	177.950	0.000	*
C	0.190	4	0.047	3.160	0.078	
Error	0.119	8	0.015			

A (hydrogen peroxide concentration), C (reaction temperature), (*p* < 0.05). * indicates statistical significance.

**Table 2:** Sensory scores for the samples in the orthogonal experiments (measured average value, *n* = 3).

Number	Factor			Score
	A (Hydrogen Peroxide Concentration)	B (Reaction Time)	C (Reaction Temperature)	
1	1	1	1	3.77
2	1	2	2	3.97
3	1	3	3	3.67
4	1	4	4	3.87
5	1	5	5	4.1
6	2	1	2	3.97
7	2	2	3	3.63
8	2	3	4	3.77
9	2	4	5	3.8
10	2	5	1	3.73
11	3	1	3	3.2
12	3	2	4	2.97
13	3	3	5	3.17
14	3	4	1	2.53
15	3	5	2	2.83
16	4	1	4	2.6
17	4	2	5	2.53
18	4	3	1	2.3
19	4	4	2	2.33
20	4	5	3	2.1
21	5	1	5	1.97
22	5	2	1	2.23
23	5	3	2	2.53
24	5	4	3	3.17
25	5	5	4	2.53
k1	3.873	3.16	3.107	
k2	3.78	3.1	3.12	
k3	3.2	3.067	3.007	
k4	2.56	3.153	3.133	
k5	2.233	3.167	3.28	
R	1.64	0.1	0.273	
Optimal level	A5	B3	C3	
Optimal combination	A_5_B_3_C_3_			
Order	R_A_ > R_C_ > R_B_			

R (Range).

**Table 3:** Verification experimental results.

Level	Factor			Score
	A (Hydrogen Peroxide Concentration)	B (Reaction Time)	C (Reaction Temperature)	
1	100	20	30	2.53
2	100	20	35	2.48

**Table 4:** Comparison of the peak area of various volatile substances.

Compound	Peak Area		Relative Percentage Content (%)
	Untreated Tuna Peptides	Treated Tuna Peptides	Reduced Percentage Due to Deodorization Treatment
Capraldehyde	1,080,182	575,277	46.7
Nonanal	1,863,713	1,029,368	44.8
Caprylaldehyde	290,207	0	100
Heptanal	217,358.5	0	100
Diethyl phthalate (background contaminant)	11,341,130.5	5,136,536	54.7
6,10-Dimethyl-5,9-undecylene-2-one	881,817	442,281	49.8

**Table 5:** Total nitrogen and amino acid nitrogen content in tuna peptide.

Sample	Total Nitrogen Content (g/100 g)	Amino Acid Nitrogen Content (g/100 g)
Untreated tuna peptides	15.4	2.48
Treated tuna peptides	13.9	3.52

**Table 6:** Sensory evaluation criteria [36].

Sensory Evaluation	Sensory Score
No fishy odor	1
Slight fishy odor	2
Moderate fishy odor	3
Heavy fishy odor	4
Very heavy fishy odor	5

**Table 7:** Factors level table of orthogonal methodology.

Level	Factor		
	A (Hydrogen Peroxide Concentration) %	B (Reaction Time) min	C (Reaction Temperature) °C
1	0.5	5	25
2	2	10	30
3	10	20	35
4	50	30	40
5	100	60	45

## Data Availability

The original data presented in the study are included in the article; further inquiries can be directed to the corresponding author.

## Supplementary Materials

The following supporting information can be downloaded at: https://www.mdpi.com/article/10.3390/molecules31040726/s1, Table S1: Tuna peptide moisturizer formula. Tables S2 and S3: Absorbance at 734 and 517 nm. Figures S1 and S2: GC-MS results of samples before and after treatment. Figure S3: GC-MS spectrum matching for 3-heptanone. Figure S4: GC-MS spectrum matching for 2-octanone. Figure S5: Quantofix Peroxide 25 test strip results. Table S4: Manganese content in tuna peptide solutions before and after H_2_O_2_ treatment measured by ICP-OES. Figure S6: Total ion chromatogram of LC-MS analysis for untreated tuna peptides. Figure S7: Total ion chromatogram of LC-MS analysis for H_2_O_2_-treated tuna peptides. Table S5: Identification and relative abundance of tuna peptides before and after H_2_O_2_ treatment. Figure S8: Calibration curve for hydrogen peroxide quantification by titanium oxysulfate method.
