# Supplementary material for: Deodorization of Tuna Peptides by Hydrogen Peroxide Oxidation
Source: Molecules. 2026 Feb 20;31(4):726. doi: 10.3390/molecules31040726 (PMC12942699; doi:10.3390/molecules31040726)
Supplement: Supplementary file 1 [file molecules-31-00726-s001.zip › molecules-4036929-supplementary.pdf]

Supporting Information

Deodorization of tuna peptides by hydrogen peroxide oxidation

Huaye Tong, Jiongfeng Li, Minjie Zheng, Xingya Fan, Wenbing Yuan, Jiangshang Su, Daofei Lv*, Feng Xu and Xin Chen*

School of Environment and Chemical Engineering, Foshan University, Foshan 528000, PR China

*Correspondence: lvdaofei@163.com (D.L.); chenxin@fosu.edu.cn (X.C.)

Table S1. Tuna peptide moisturizer formula

Item number	Ingredient name	Mass percentage (%)
1	Water	43.4
2	Glycerin	6
3	Propylene glycol	4
4	Butylene glycol	4
5	Sodium hyaluronate	0.1
6	Disodium EDTA	0.1
7	Allantoin	0.1
8	Methylparaben	0.2
9	Tuna peptide	0 or 1
10	Cetearyl alcohol	2
11	Emulgade 165 CN	1
12	Emulgade PL 68/50	1.2
13	GTCC	12
14	Dimethicone	2
15	IPM	2
16	Ethylparaben	0.05
17	Propylparaben	0.05
18	Tocopheryl acetate	0.2
19	Olive oil	0.5
20	BHT	0.05

Table S2. Absorbance at 734 nm

	**Experiment 1**	**Experiment 2**	**Experiment 3**
A_0	0.519	0.514	0.520
A_1(original tuna peptide)	0.007	0.006	0.009
A_2(H_2O_2-treated peptide)	0.043	0.043	0.045

Table S3. Absorbance at 517 nm

	Experiment 1	Experiment 2	Experiment 3
A_0	0.210	0.220	0.190
A_1(original tuna peptide)	0.030	0.029	0.031
A_2(H_2O_2-treated peptide)	0.456	0.455	0.457

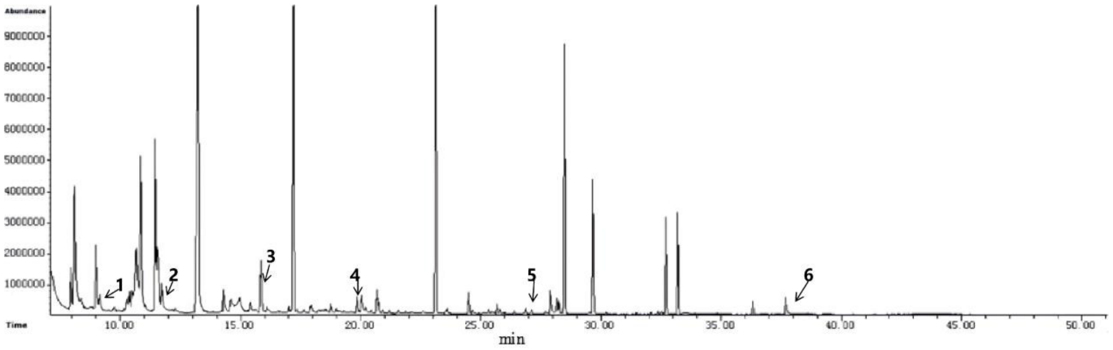

Figure S1. Total ion chromatogram (TIC) of GC-MS analysis for untreated tuna peptide solution by SPME extraction. 1. Heptanal, 2. Caprylaldehyde, 3. Nonanal, 4. Capraldehyde, 5. 6,10-Dimethyl-5,9-undecylene-2-one, 6. Diethyl phthalate.

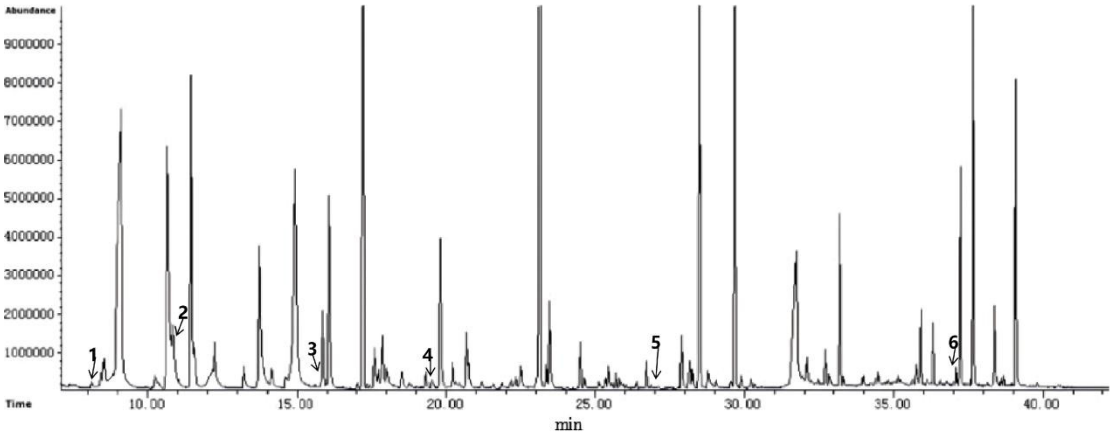

Figure S2. Total ion chromatogram (TIC) of GC-MS analysis for a tuna peptide solution deodorized with hydrogen peroxide using SPME extraction. 1. 3-Heptanal, 2. 2-Octanone, 3. Nonanal, 4. Capraldehyde, 5. 6,10-Dimethyl-5,9-undecylene-2-one, 6. Diethyl phthalate.

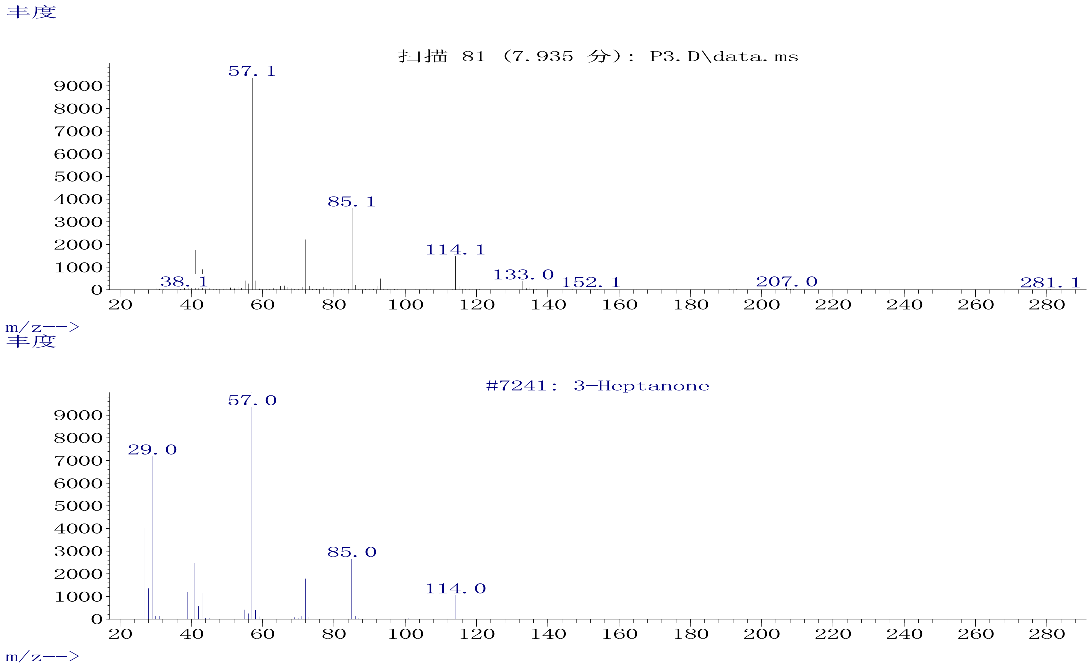

Figure S3. GC-MS spectrum matching for 3-heptanone.

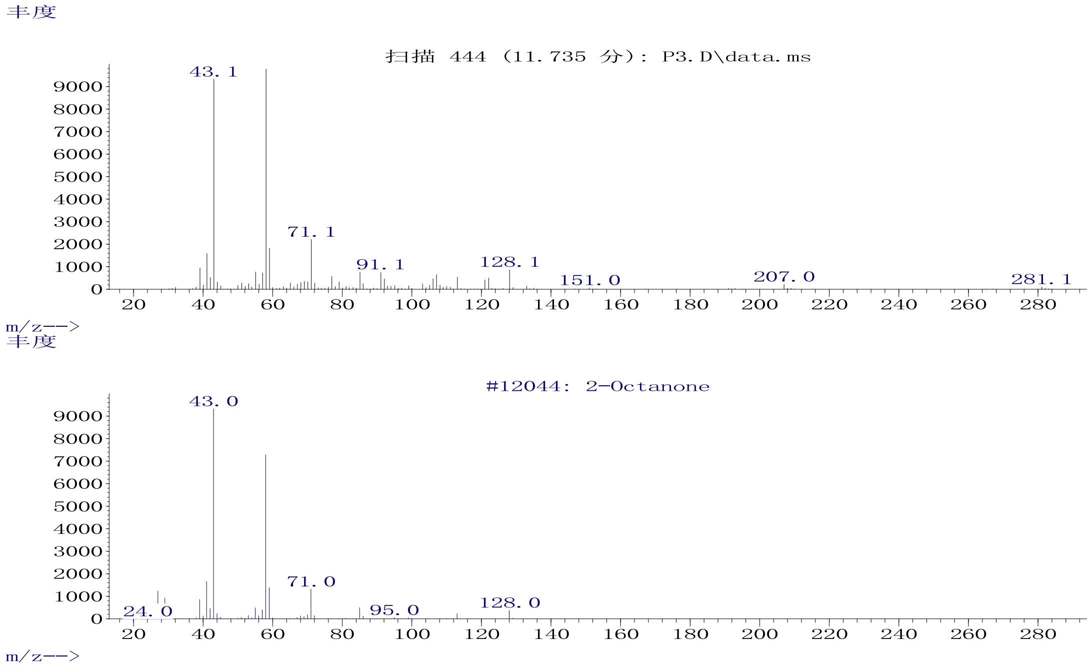

Figure S4. GC-MS spectrum matching for 2-octanone.

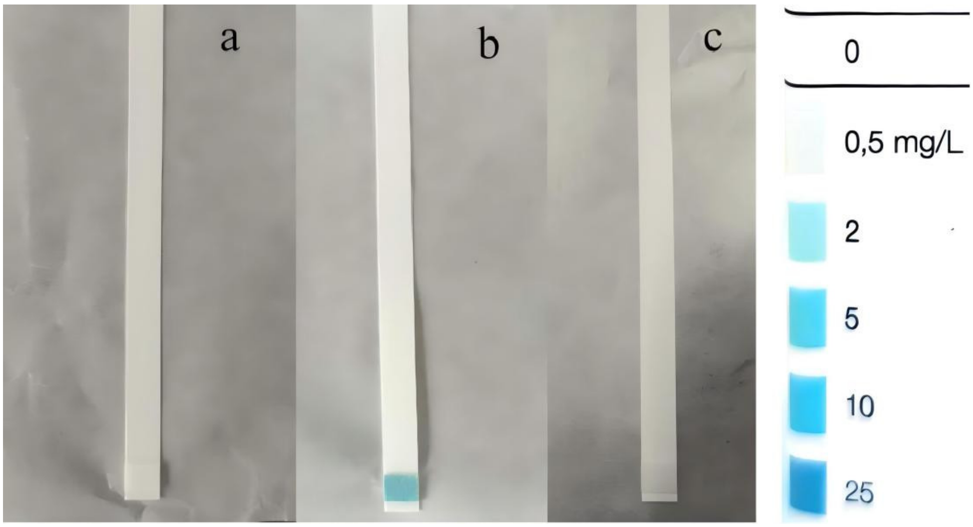

Figure S5. Quantofix Peroxide 25 test strip detection results. a. water, b.H_2O_2-treated tuna peptide without adding MnO_2, c. H_2O_2-treated tuna peptide with adding MnO_2, d. standard color comparison card for hydrogen peroxide solutions of different concentrations.

Table S4. Manganese (Mn) content in tuna peptide solutions before and after hydrogen peroxide treatment.

Sample	Sampling Volume (mL)	Dilution to Volume (mL)	Element Analyzed	Element Concentration in Test Solution (μg/L)	Dilution Factor	Element Concentration in Digestate/Original Sample Solution (μg/L)	Element Content in Sample (mg/L)
Before Treatment	1	10	Mn	3.7207	1	3.7207	0.04
	1	10	Mn	3.6259	1	3.6259	0.04
	1	10	Mn	3.6974	1	3.6974	0.04
After Treatment	1	10	Mn	1148.2000	1	1148.2000	11.48
	1	10	Mn	1492.2000	1	1492.2000	14.92
	1	10	Mn	1322.8000	1	1322.8000	13.23

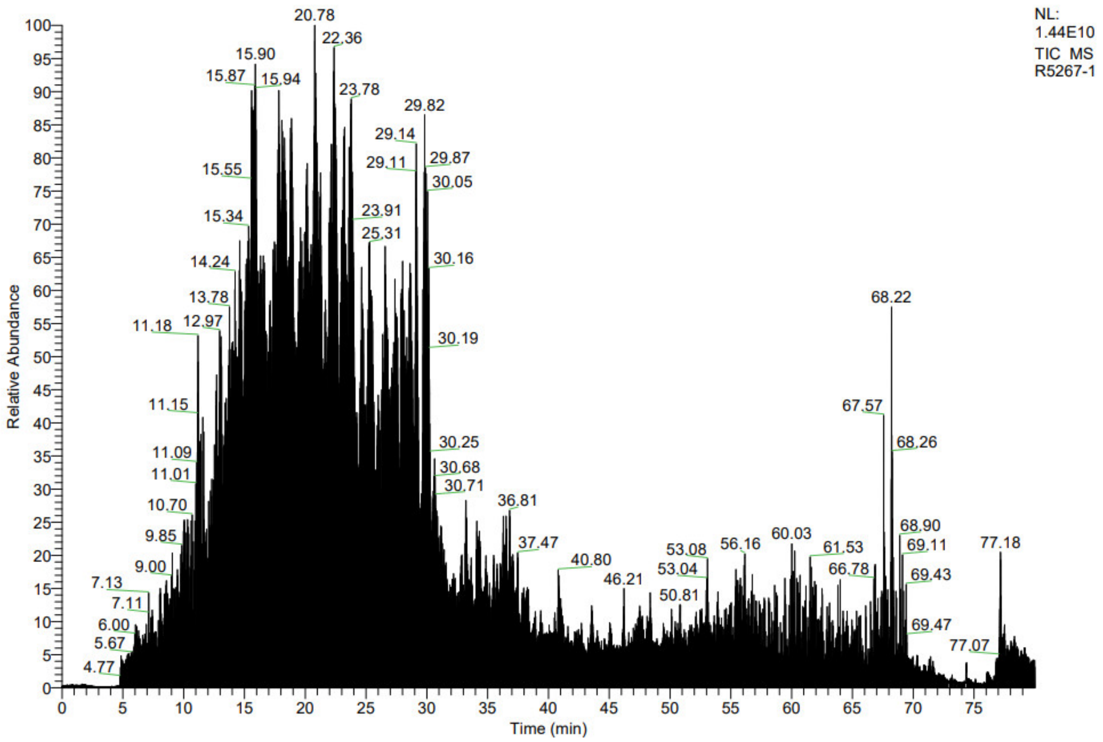

Figure S6. Total ion chromatogram (TIC) of LC-MS analysis for untreated tuna peptide.

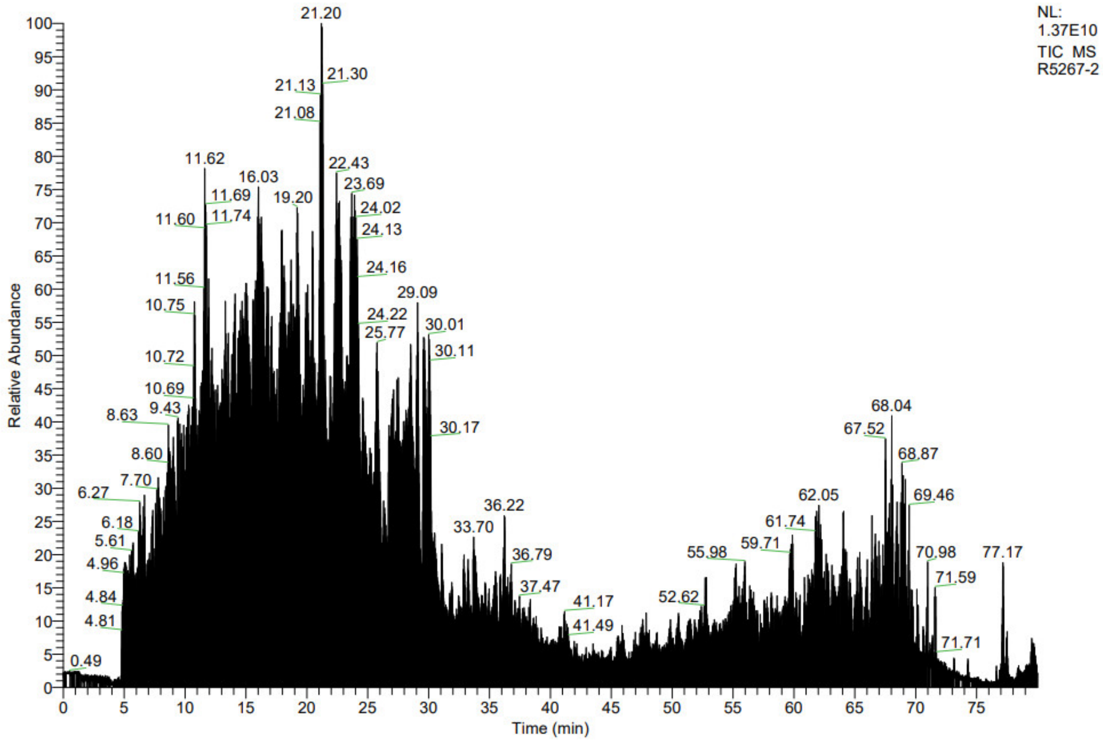

Figure S7. Total ion chromatogram (TIC) of LC-MS analysis for a tuna peptide solution deodorized with hydrogen peroxide.

Table S5. Identification and relative abundance of tuna peptides before and after

hydrogen peroxide treatment.

Sequence	Before Treatment	After Treatment	Intensity 1	Intensity 2
KREKVLKLLLINEC	1		0	0
QDFAQFADHLG	1	1	12308000	11622000
SATGNTILGANVFNV		1	0	0
LHLFTWRWQ	1		0	0
SRIIKYLKEMEDGQS	1		0	0
QLAYLRELMKLMLPGFWK	1		0	0
CYWFNPNSFETD	1		0	0
YHLILDWKSQPVIEKVGLE		1	0	0
PRPPWTIHDHHDHHHHAEEHH	1		0	0
TNDEEEYQKYVKMLQLKELIFKH	1		0	0
KKLQMEYMQRQEKLEGDQKNMA		1	0	73730000
SPFTTMQFFDGSPFQINDEYCK	1		98227000	0
DFDHSCQRETPSVVAMIYPFVG	1	1	20379000	26642000
FNNFRVQFETTSSGQECSETCL	1		112950000	0
PDFFSDDNHEVSGSDEDDDPPL	2	1	245500000	62178000
SFCMSMWYHMSGESIGTLTLYQ	1		81006000	0
ADRHFCPDLQFCYRLYYNIFRK	1		30773000	0
CFIPIFYEYEITSTNEYLERRF	1	1	26022000	40659000
DSCGYACTFEEDICGWTIGDET	1	1	122850000	94924000
GVMDASTDTLFDGVEGEESADM		1	0	216790000
KETSHYSKVKDDDSDSDDSDEE	1	2	130150000	395810000
LEDLECHQESQMVPHRQPRHPR	1	1	81391000	79499000
MWLLSYMVIHPDIQDRVHEELD	1		208550000	0
QMRRYVPLRYGKDQFKDIKYDY		1	0	120150000
TASSYYSVYHLPDHARLHYEYY		1	0	33397000
YCRFQKCLRMGMMLEAVREDRM	1		139130000	0
ACSWDNAQYGDEFDWFRHSGGT		1	0	20029000
ADNESSYNTVECYSPAEDKWRY	1		180910000	0
CPHEGWGTHNCQHYEDASVACT		1	0	57204000
CRDGTCDGCNFLILWNTEMACP	1	1	128260000	225650000
CSDSSSEWTNSWNSQDHHKKGE		1	0	219620000
DCGCSGRTTSCTYDEDKGYGVC		1	0	29311000
DCNYYNLDKVKSSCKWDEEEQN	1	1	68009000	205330000
EEKNEVTQENESDDWVKDNEKV	1		238320000	0
ETSRDVFEEELRLRDEALAEME	1		32057000	0
FQSFYCHDGTSQCFGKLDYDTS	1	1	223350000	219620000
GVYWYESVEQKDPFIKFENDEI	2	1	733280000	150800000
IPPQDREAYFRYYLSFFHHEQV		1	0	46084000
LDGQNNMDNEEDDDLETPENET	1		140460000	0
MFHQVRVSTKDCDALRFLWWRD	1		71702000	0
MIYWVDTEEQKIYRRKRDHTGK		1	0	72332000
QQFDRTNFSYDVNRQLEKMLYI		1	0	71824000
QSELDFLNQLHMDRYGETGYKC	1	1	81915000	23245000
REKLQCKSFKWYHENVYPELRL	1		245800000	0
RHTMIQETREQSDQEDDENGEE	1		131180000	0
RKAEYFGIPEEKRFIYKRYMFE	1		33356000	0
SNRIFCYYYQVLTEYASRQTRS	1		67209000	0
TDNSTHSFYSNTVVNHDNNTNM	1	2	798600000	933610000
VNQWRNTSSVIEWFKEHTRQRE	1		132350000	0
VTAKPQNRQCREYSDSGYEESD		1	0	40812000
WKFHRYELIMEYSERPCLPPPL	1		99927000	0
FNNELQVYTAASSIHGCHADAD	1		24878000	0

Sequence	Before Treatment	After Treatment	Intensity 1	Intensity 2
ELYGADFMMTDDFDPWLIEINC	1	1	71012000	364220000
AALEEEKEGFQETAENDFERDQ		1	0	100630000
QCDPSTGMCLNCQFRTEGFNCE	1	1	25953000	117810000
EWRDLKIVYKRYASLYFCCAIE	1	1	74914000	54414000
CNDLSKQPWHCSNDYKTYYANA		1	0	51343000
IHEEYASNYKRAFTTLKEEKEE	1		110240000	0
KFTSQMELLRRSRMSPFYDFGP	1		24122000	0
KTIERIRDTFYWSGYRRHVEKW	1		96903000	0
PSSEQFEEFLPLFLDDVPNEIC	1	1	72849000	63762000
QTDRQKERKKKREREREKERQT	1	1	106730000	82306000
RSSTRYLTEDPKDTVKMTQIFI		1	0	27438000
VVIQCMEPQNGASPPYIFDQWS	1	2	91704000	92464000
PCEDGFSGPGCLQRCRCRNEAT	1	1	45642000	44511000
PNGEIREYHVYEDEYLITPIYT	1		73756000	0
EQWRIYHQIVVPRTYRREILTI	1	1	70333000	80789000
ESSLSEVDDMMLAGHLRLARVL	1	1	16270000	10357000
HDFRWIMDAFIRSSVEYEQNVG	1	2	66208000	78310000
IAREEWKQKEKERLELENRRIL		1	0	51212000
INSHLAAHLEEYERRNQDYNDI		1	0	19842000
KQFSRLNLRKYFFSQRVVDIWN		1	0	89562000
LRIDPYSDYETFLDRVRRFTYT		1	0	29368000
NGSFIHINQSWRVNCQYAEMED	1	1	222190000	255960000
QFYKEYPEYERLCKDIMRVTEL	1		36504000	0
QKREQERARLEREKQREREEEE	1		105990000	0
RRQIRRQRGLESDDEEEDEKKD	1	1	64318000	95191000
TFMSVLERYLPYWMGSVMFQWT	1		30272000	0
VLRKRRQRIKSEANWELFYHRF		1	0	82708000
WPFSGLRRWWRRWNYPPVRLLS	1	1	234430000	182700000
YDGWSSYDCAEGHRGAWWFAKQ	1		6388300	0
YKGNKFIHYIDGFDFEKSNWMR	1		33634000	0
AYYGEGNGPILYDIDCSGWEDS		1	0	112390000
CDGYEMSLAECQTNIEMESPQS	1	1	59916000	446790000
CDTRIEACRCPKSYSGHDCSMC	1	2	561310000	163950000
DSDDASPTSMFFDFVTNSDDSE	1		47924000	0
SDGYELDEDQRSCIDIDECSSS		1	0	73671000
SEDEMSEGEDDYFKIPEARNPS	1	2	110490000	1153700000
YVDDDEDSESDPEENEIHLLGI	1	1	45725000	81978000
AEQAHLWAELVFLYDKYEEFDN		1	0	167020000
CRTCSHNCRDCANSYLQCISCN		1	0	34152000
DDDDDDNNDSKREETTATKYED	1	1	9844600	63452000
DDDDDDTIYLKCTESEFHKHRI		1	0	54748000
DEQQAQTQHQPWWKANFFIREP	1		78724000	0
DNQMEMEGRPSSDEESSSDDDK	1		42898000	0
DRTKIYQIDDGGEMEMTECVNP		1	0	184640000
EDNRDGINSDGRNSDDDVDDDD	1	1	291590000	1059200000
FLHIDELAEWRVYRDWLRFWTH		1	0	50853000
FVLKHQLQWKKHMEWFNKEVSA		1	0	34759000
HQECLVQWLKHSKKEYCELCMH	1		30272000	0
KHKNSRFYEAEVIGEREQMFCH	1	1	84361000	79208000
KMYDFLRREKEKEIQGVQEKYD		1	0	50961000
LALIHDRNEFDRFEKERVQVRW		1	0	120980000
MFEKEKKLTEMKAEQFRMENEM		1	0	28287000
MTQSNRMTTSQNNMMTSQSNMM	1		27208000	0
NDAHSGNCAANYRAGWWYKSCN		1	0	37669000
NPNPCQNGGICDDLNRNYSCMC	1		105910000	0
PGVEGDAQETQEEEEEDDEDDK	1		3733600	0

Sequence	Before Treatment	After Treatment	Intensity 1	Intensity 2
QTLERKYGKHMNIDMFNLIDIY	1		124020000	0
RPSSSASRQAWNDNEGFDMGSE		1	0	24221000
TNYNFDIMHDMLEGVCPFEVKL	1		170630000	0
ACDDYISCDFERGICDWEQIHT	1		0	0
CKERRRYRRNAQVLDPFDLQYL	1	1	20815000	19043000
EGWRLSWRRVLYVHWDRAEYML	1		487880000	0
KIEEEKKVLIEKMDKMKAEFES	1		26285000	0
REQYFDCFNSSTNLTEMMKTRR		1	0	89418000
WDDLWVRDGRIEDPRKLFWEER	1		5372700	0
DGSPFSYTDWYSRETMYLEFQP	2	1	70135000	85638000

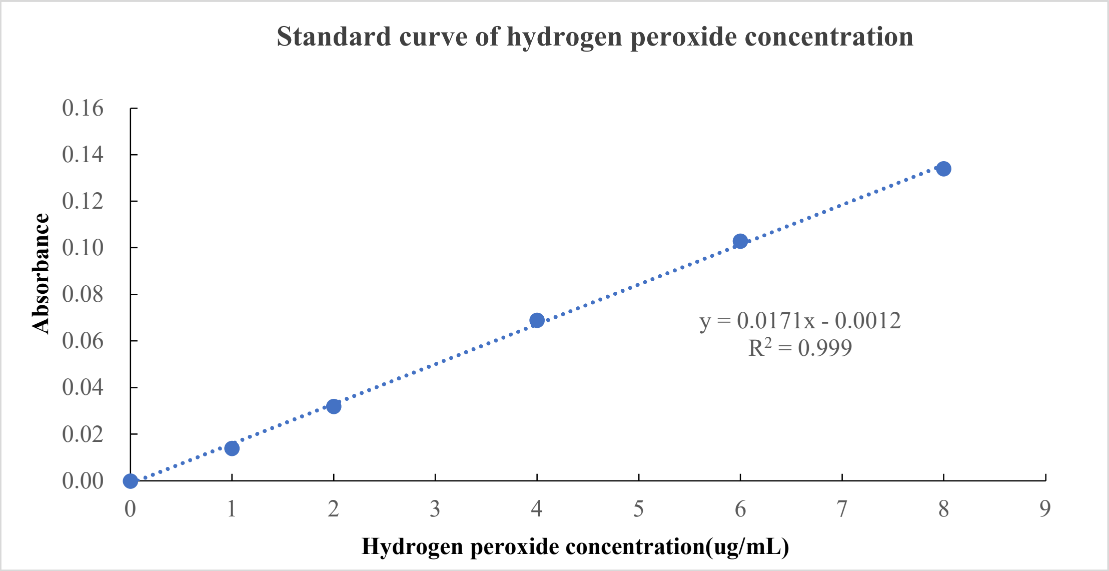

Figure S8. Calibration curve for hydrogen peroxide concentration.
